# Using Sensory Cues to Optimise the Satiety Value of a Reduced-Calorie Product Labelled ‘Healthier Choice’

**DOI:** 10.3390/nu12010107

**Published:** 2019-12-30

**Authors:** Keri McCrickerd, Priscilla Pei Sian Tay, Claudia Shuning Tang, Ciarán Gerard Forde

**Affiliations:** 1 Clinical Nutrition Research Centre (CNRC), Singapore Institute for Clinical Sciences (SICS), A*STAR Research Entities and National University Health System, Singapore 117599, Singapore; 2 Department of Physiology, Yong Loo Lin School of Medicine, National University of Singapore, Singapore 117593, Singapore

**Keywords:** health labelling, calorie reduction, satiety, eating behaviour, sensory evaluation

## Abstract

Reformulation strategies to reduce the energy density of commonly consumed foods and beverages are intended to support weight management, but expectations generated by labelling these as ‘healthier’ alternatives can have unintended effects on the product’s sensory evaluations and consumption behaviours. We compared the impact of four different strategies for presenting a lower-calorie beverage to consumers on product perceptions, short-term appetite and energy intake. Participants (*N* = 112) consumed higher- (211 kcal/portion) and lower-calorie (98 kcal/portion) fixed-portion soymilks in the morning across two test days, with the lower-calorie version presented in one of four contexts varying in label information and sensory quality: (1) sensory-matched/unlabelled, (2) sensory-matched/labelled, (3) sensory-reduced (less sweet and creamy)/labelled, and (4) sensory-enhanced (sweeter and creamier)/labelled. The label was Singapore’s Healthier Choice Symbol, which also highlighted that the soymilk was lower calorie. Changes in reported appetite, ad libitum lunch intake, and self-reported intake for the rest of the text day were recorded. Results indicated that total energy intake was consistently lower on the days the lower calorie beverages were consumed, regardless of how they were presented. However, the ‘healthier choice’ label increased hunger prior to lunch and reduced the soymilks’ perceived thickness and sweetness compared to the same unlabelled version. Increasing the product’s sensory intensity successfully maintained liking, experienced sensory quality and appetite. Results suggest that food companies wanting to explicitly label product reformulations could combine messages of ‘lower calorie’ and ‘healthier choice’ with appropriate taste and texture enhancements to maintain acceptance and avoid negative effects on appetite.

## 1. Introduction

The increasing global prevalence of overweight, obesity and related cardio-metabolic diseases requires changes to the current food environment. One strategy is to use a combination of calorie reductions and downsized portions to reduce the energy content (though not necessarily the nutrient density) of commonly consumed foods and beverages [1,2,3,4], and offset what has been estimated as a 100 kcal daily energy intake surplus that is driving population-based weight gain in countries such as the UK and USA [5,6]. To achieve this, the food industry must consider how reformulations are presented to the consumer to optimise their role in diet and weight management.

The success of reduced calorie products for weight management relies on consumers (i) accepting and using these products and (ii) not compensating for reductions in energy intake by eating more at a later time [7]. Reformulation strategies often aim to generate products that look and taste like the ‘original’ versions but contain fewer calories, so consumers can reduce their energy intake with minimal changes to their usual eating habits [8] or product sales [9]. A common example is the use of low-calorie sweeteners (LCS) to replace some of the calories from sugar in soft drinks and sweetened dairy products [10]. These products have been shown to reliably reduce energy intake and body weight when consumed in place of higher-calorie sugar-sweetened versions across periods of 10 weeks to 40 months [11]. Reduced energy intake occurs because variations to the energy content of one meal or snack are rarely fully compensated for at later eating occasions [12], particularly when consumers are unaware of the caloric differences between products [13,14,15].

A practical challenge to implementing ‘silent’ calorie reductions to popular items is that food companies are typically incentivised to promote the benefits of their products to increasingly health-conscious consumers [9,16]. As a result, people are often informed when a product is ‘lower calorie’, contains ‘less fat’ or has ‘reduced sugar’ through front-of-pack (FOP) labels, and this can modify beliefs about these products even before they are consumed. Label-driven expectations of how a food or beverage should look and taste guide the actual experience of its flavour quality, and influence broader beliefs about its health and monetary value [17]. For example, people perceived the same sausage to be less salty and fatty when it was labelled as ‘lower fat’ [18], and judged instant noodles labelled as ‘reduced MSG’ to be healthier and lower calorie than the same ‘original’ version [19]. Believing a food is ‘healthier’ can result in an underestimation of its energy content [20], reduced expected [19] and experienced feelings of fullness [21] and increased consumption of that food [22] or others at later meals [23,24,25,26].

The influence of ‘reduced calorie’ or ‘healthier’ labels on consumer perceptions and behaviours could be partly due to the increased salience of these cues when all other features of the reformulated product’s taste, texture and appearance are kept the same. Recent evidence suggests that systematically varying the flavour intensity of a food or beverage product had a larger impact on consumers perception of fullness and portion size decisions than the addition of FOP labels, including ‘reduced sugar’ and ‘healthier choice’, even when these labels led consumers to believe that the product was healthier and lower in calories [19]. This suggests that sensory modifications could be used to orient consumers to the potential satiating power of foods, which may be compromised by beliefs generated by health and nutrient labels [27]. However, concerns have been raised over possible negative effects of a mismatch between expectations generated by how a food or beverage looks and tastes and how it is experienced upon consumption, both for the product’s acceptance and sensory appraisal [17] and the likelihood of experiencing rebound hunger [28].

As the optimal way to present reduced calorie foods and beverages to consumers is unclear, we aimed to test the efficacy of the different approaches on product evaluations, short-term appetite and energy intake. Participants evaluated and consumed higher (‘Original’) and lower (‘Reduced Calorie’) energy density beverages across two test days, with the reduced calorie versions presented in one of four contexts varying in labelling and sensory intensity: (1) sensory-matched and unlabelled, (2) sensory-matched and labelled, (3) sensory-reduced and labelled, and (4) sensory-enhanced and labelled. The sensory-matched contexts were designed to represent a typical reformulation strategy where products are made to look and taste like the original versions, and the reformation can be ‘silent’ (unlabelled) or explicitly promoted FOP. The sensory-reduced and sensory-enhanced contexts were designed to represent taste and texture modifications that could be used to orient consumer perceptions towards (sensory-reduced) or away from (sensory-enhanced) those suggested by the labelled FOP health message. The label selected was Singapore’s Healthier Choice Symbol [29], which is heavily promoted by public health bodies in Singapore and food companies can apply to have it attached to qualifying products. We recently showed that applying the symbol to popular food and beverage products increased their perceived healthiness and monetary value, but also lead to underestimations of the calories in a serving [19], underscoring the importance of understanding how such labelling systems are likely to impact the acceptance of reformulations, as well as potential changes to appetite and energy intake.

## 2. Materials and Methods

### 2.1. Design

A 2 × 4 mixed design assessed the effect of consuming a fixed-portion caloric beverage varying in Energy Content (higher and lower energy density; measured within-subjects) on product perceptions, short-term appetite and energy intake when the lower calorie version was presented in one of four Contexts varying in label and sensory cues: (1) sensory-matched unlabelled, (2) sensory-matched labelled, (3) sensory-reduced labelled, and (4) sensory-enhanced labelled (measured between-subjects). Participants were randomised into one of the four beverage context groups (single blind) and consumed the original and reduced calorie beverage across two non-consecutive test days. A randomisation schedule was generated in excel by one of the research team, and participants were assigned upon recruitment by another team member. The order in which the higher and lower energy soymilks were presented was fully counterbalanced within each group. Primary outcomes were the sensory appraisal of the beverages and energy intake at a later laboratory meal depending on the beverages’ energy content and context. Changes in rated appetite and self-reported energy intake outside of the laboratory were considered as secondary outcomes. A power calculation (G*Power) using a two-way alpha of 0.05 and a within-groups correlation of *r* ≤ 0.7, indicated that a minimum of 24 participants per group would be required to detect a 2 × 4 interaction (medium effect size, *n_p_*^2^ = 0.12) between the beverage energy content (within-groups) and context (between-groups) on later energy intake. The parameters were based on a previous study testing a similar effect of consuming higher and lower energy beverages varying in sensory quality and the belief that it would be ‘thirst-quenching’ or ‘filling’ on later energy intake in a young adult population [30], which suggested a 29% increase in responsivity to the energy difference (signified by changes to energy intake at later meal) as the beverage context became more satiety-relevant. The research was granted ethical approval by the Singapore National University Health Domain Specific Review Board and conducted in accordance with the Declaration of Helsinki.

### 2.2. Participants

One hundred and twelve participants (n = 28 per group) were recruited to take part in a study investigating the link between “Reduced Calorie Food and Mood”. Eligible participants were males and females aged 21 to 50 years old with a body mass index (BMI) between 18.5 and 30.0 kg/m^2^, without any allergies or aversions to the study foods and ingredients. Participants were not eligible to take part if they were actively dieting, smoked >7 cigarettes per week, had experienced weight gain/loss of more than 5 kg in the last 12 months, or taking medications known to affect appetite or metabolism.

### 2.3. Test Foods

#### 2.3.1. Standard Breakfast

Participants received a standard breakfast at the beginning of each test day, consisting of a test beverage (described below), whole apple (China Fuji Apple, Medium size) and cereal bars (Nutrigrain Soft & Fruity Bar, Kellogg’s, Manchester, UK). Breakfast provided females 32% and males 34% of the daily Recommended Dietary Allowances (RDA) for energy intake when the original soymilk was served, and 23% and 24% respectively and when the reduced-calorie soymilk was served. The RDA were based on recommendations from the Health Promotion Board, Singapore [31].

#### 2.3.2. Test Beverages

**Figure 1:**
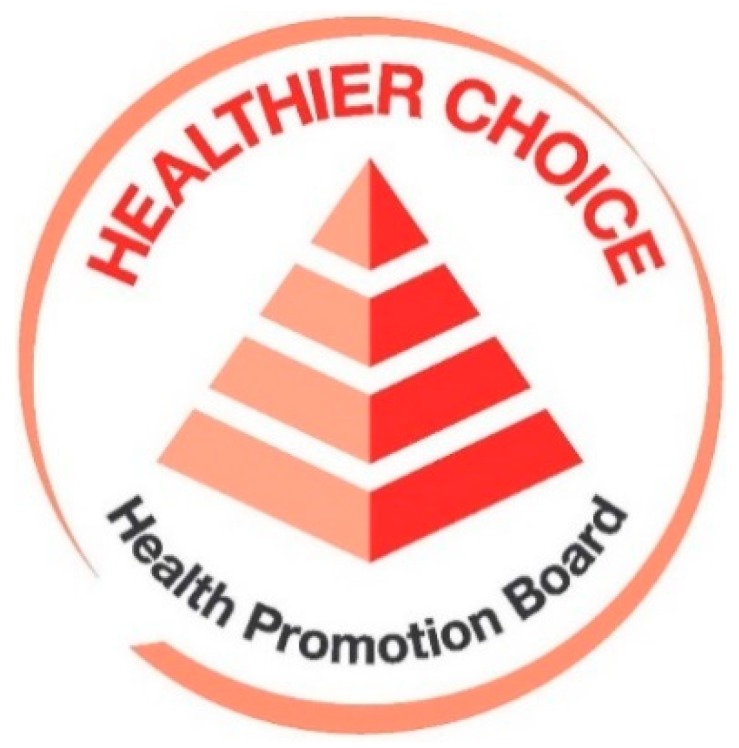
The ‘Healthier Choice Symbol’ created by Singapore’s Health Promotion Board [29].

**Figure A1:**
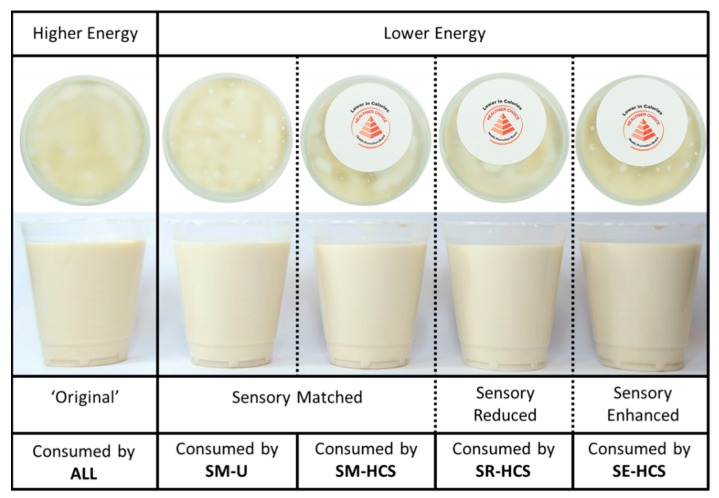
Soymilk Assignment and Images.

A higher energy (211 kcal per 315 mL portion) and lower energy (98 kcal per 315 mL portion) soymilk were developed in-house based on a recipe described previously [14]. Full recipes and images of the test products are provided in Appendix A Table A1 and Figure A1. The soymilks consisted of a base made from 250 g Nutrisoy No Added Sugar soymilk (F&N Foods Pte Ltd., Singapore) with varying quantities of Maltodextrin (MALTRIN^®^ M100, Singapore: DE 10-12), guar gum (Myprotein^®^, The Hut Group, Manchester, UK), sucralose (Heartland Food Products Group, Carmel, IN, USA), vanilla extract (Nielsen-Massey Vanillas Inc., Waukegan, IL, USA) and cream cheese flavouring (LorAnn Oils, Lansing, MI, USA), which were used to vary the soymilks energy density and sensory quality. The sensory characteristics of the original soymilk were modelled on a commercially available product (Nutrisoy ‘Original Fresh Soya Milk’; F&N Foods Pte Ltd., Singapore), and the sensory characteristics of the reduced-calorie versions were varied across the context groups and presented with and without the Healthier Choice Symbol (HCS; Figure 1) as follows:

- *Sensory-matched unlabelled (SM-U)*—Sensory matched to taste like the original (equally sweet, thick and creamy), without a label identifier (covert energy reduction).
- *Sensory-matched labelled (SM-HCS)*—The same sensory matched beverage as above, but with the HCS attached (explicit energy reduction).
- *Sensory-reduced labelled (SR-HCS)*—Designed to taste less thick, sweet and creamy than the original, with the HCS attached (explicit energy reduction).
- *Sensory-enhanced labelled (SE-HCS)*—Designed to taste thicker, creamier and sweeter than the original, with the HCS attached (explicit energy reduction).

All participants were told that seeing an HCS signalled that they were consuming a reduced-calorie soymilk. This meant that only participants in the SM-U condition were unaware when they consumed the lower calorie soymilks, as it was not labelled. Our previous research indicated that soymilk was considered as neither heathy or unhealthy, with a mean rating of 49 ± 3 on a 100-point VAS (where 0 represented the least healthy a product could be), while the addition of the HCS significantly increased its perceived healthiness and reduced its expected calorie content [19].

#### 2.3.3. Ad Libitum Lunch

Lunch consisted of 800 g Yang Zhou Fried Rice (1.27 kcal/g; JR Foods, Singapore) and a 250 mL glass of water. The main ingredients included rice, egg, chicken char siew, shrimp, cabbage, carrot and vegetable oil. Participants were instructed to serve themselves as much as they liked and eat until they were comfortably full. Refills of food and water were available, but no participant consumed the entire food serving.

### 2.4. Procedure

Potential participants attended a screening session to assess their eligibility and provide baseline measures of dietary restraint [32], familiarity with the HCS, height (cm) and weight (kg) and body fat percentage determined using a Tanita BC-418 Bioelectric Impedance Analyser.

**Figure A2:**
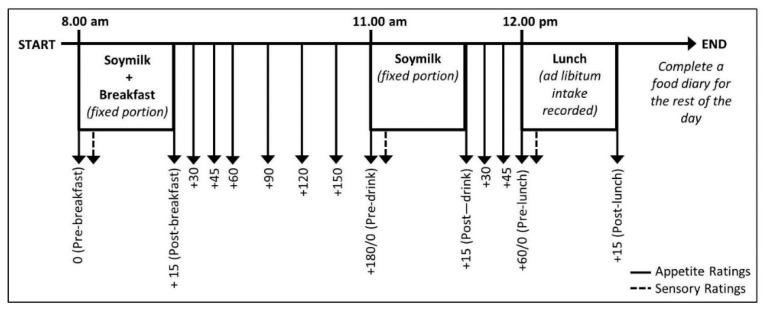
General Procedure for the Test Days.

The test day procedure is summarised in Appendix A Figure A2. On arrival to the Clinical Nutrition Research Centre (CNRC) between 8.00 and 9.00 am, participants verbally confirmed that they had fasted from the evening before and refrained from engaging in vigorous physical activities. Participants began by completing a set of computerised appetite ratings disguised as “Mood Questions” (pre-breakfast rating), which involved rating the extent to which they felt hungry and full, their desire to eat and prospective consumption using a 100-point Visual Analogue Scale (VAS), alongside ratings of happy, alert and clear-headed as distractor “mood” questions. All questions were made in a randomised order.

Participants were then provided with breakfast and instructed to taste a mouthful of the soymilk beverage using the straw provided and rate how sweet, thick, creamy, pleasant, filling and familiar it was, following the same randomised VAS format as the appetite ratings. Participants were given 15 min to consume breakfast before completing a second set of appetite ratings (post-breakfast rating/+15 min). Participants were then able to leave the CNRC and continued to complete appetite ratings at 15- or 30-min intervals for the remaining 2 h, prompted with reminders set on their mobile device. Participants were not permitted to consume any food in this time and recorded all water consumption in a food diary. Participants returned to the CNRC mid-morning to consume another portion of the soymilk as a snack and continued to rate their appetite pre- and post-drink and at 15 min intervals before returning for lunch 1 h later.

At lunch, participants were instructed to taste a mouthful of the fried rice and rate how pleasant, and familiar it was. Participants then consumed lunch and rated their appetite one final time (post-lunch rating). Total lunch intake was recorded to 0.01 g using a Sartorius balance. Food and beverage intake (including water) for the rest of the day was recorded in a food diary which was entered using nutrient databases (FoodWorks 8.0.3553; Xyris Software, Australia) and analysed by a single trained researcher. At the end of the second test session, participants were asked to identify what they thought was the purpose of the study and reimbursed S$60 for their time.

### 2.5. Data Analysis

In the first instance, a series of between-groups ANOVAs were used to compare participant characteristics across the four Context groups. Separate 2 × 4 Mixed Linear Models were then used to assess the effect of soymilk Energy Content (within-subjects) and *Context* (between-subjects) on the outcomes of interest: sensory and hedonic evaluations, changes in rated appetite, ad libitum lunch intake (kcal), intake reported in the food diaries (kcal) and cumulative energy intake over the course of the day (kcal). Sex and order the beverages were presented in had no influence on the main outcomes so these variables were removed from the final statistical models. The additional within-subject variable of Time (13 time points from pre- breakfast to post-lunch) was included to test for changes in rated appetite, controlling for baseline appetite ratings on each day.

Participants who reported an increased tendency to clear their plate (r = 0.378, *p* < 0.001) and higher dietary restraint (r = −0.279, *p* = 0.003) ate significantly more across all the intake outcomes, and therefore these variables were included as covariates in analyses of intake and appetite. No other significant covariates were identified. One male participant (in the SM-HCS group) showed intake scores > 3 SD from the mean that strongly skewed the data and could not be corrected with transforming. Thus, the data from this participant were removed from the final analyses, which were based on data from n = 111 participants. Estimated Means (M) are presented alongside 95% Confidence Intervals (±CI) throughout unless otherwise stated. Bonferroni corrected comparisons were used to interpret any significant main effects of beverage context or interactions. All analyses were performed in SPSS version 23 and *p* < 0.05 was considered a statistically significant difference.

## 3. Results

### 3.1. Participant Characteristics

Sample characteristics are presented in Table 1. Participants in the four beverage context groups were similar in age, percentage body fat, dietary restraint and frequency of consuming the study foods. All participants were able to correctly identify the HCS and more than half in each group reported having purposely used the HCS to make a food choice in the past. No participants correctly identified the purpose of the study.

### 3.2. Sensory and Hedonic Ratings

Sensory and hedonic evaluations of the test soymilks and lunch are presented in Table 2. There was a significant Energy Content × Beverage Context interaction across all of the ratings, which indicated that perceived differences between the original and reduced-calorie soymilks depended on the beverage context in which they were presented. Specifically, the soymilks were rated as similarly pleasant, sweet, thick, creamy and familiar in the sensory-matched unlabeled context (SM-U). However, the same sensory-matched reduced-calorie soymilk was rated as less sweet and less thick than the original version when it was presented with the HCS. The sensory reduced soymilk was rated as less pleasant, sweet, thick and creamy than the original version, while the sensory enhanced version was rated as equally pleasant, significantly creamier and less familiar than the Original version, but not significantly sweeter or thicker (although the means were trending in this direction). All the drinks were expected to be equally filling, regardless of their energy content or context. Participants rated the test lunch as equally pleasant and familiar across each of the test days, regardless of the beverage context group in which they were presented. 

### 3.3. Energy Intake at Lunch and for the Rest of the Day

**Figure 2:**
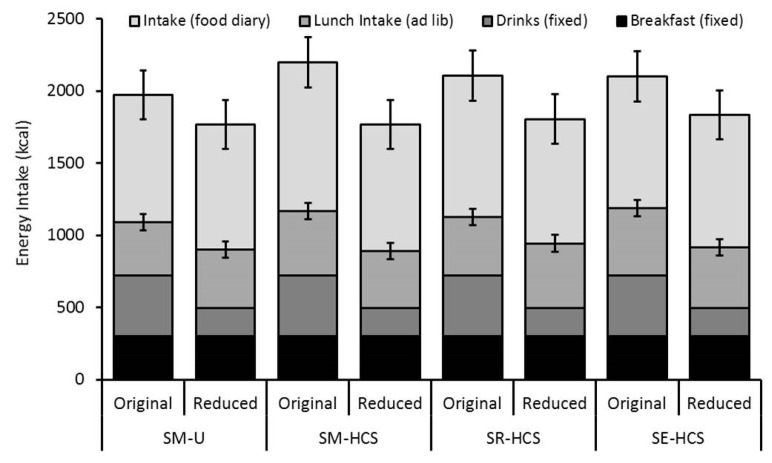
Mean (±95% CI) energy intake across the test day from the fixed breakfast, test drinks, ad libitum lunch intake and food diary records in response to the Original and Reduced calorie beverages consumed in the four contexts. The raw means are presented in Appendix A Table A2.

Energy intake is reported in Figure 2. Overall, there was a significant main effect of beverage Energy Content on lunch intake (F (1, 105) = 10.01, *p* = 0.002), with participants consuming significantly more at lunch on the days the lower-calorie beverages were consumed (M = 436.7 ± 28.2 kcal) compared to the days they consumed the higher-calorie beverages (M = 403.7 ± 28.2 kcal), independent of the beverage context group. This adjustment was small, however, and only accounted for 16% (33 kcal) of the energy intake deficit (226 kcal) that was achieved through consuming the lower-calorie drinks in place of the higher-calorie ‘original’ versions. However, there was no effect of beverage Context on lunch intake (F (3, 105) = 1.15, *p* = 0.334), and no interaction with Energy Content (F (3, 105) = 0.24, *p* = 0.869), indicating that participants consumed a similar amount of lunch regardless of the soymilk’s sensory and labelled information.

There was also no significant effect of Energy Content or beverage Context on food intake reported in the food diaries for the rest of the day (F ≤ 1.96, *p* ≥ 0.164 for both main effects and interaction). This means that participants made no further adjustments to their food intake away from the laboratory. As such, cumulative daily energy intake (including breakfast, test soymilks, laboratory lunch and self-reported intake) was significantly lower on the days the lower-energy soymilks were consumed (M = 1812 ± 95 kcal) compared to the higher-energy versions (M = 2075 ± 94 kcal; main effect of Energy Content: F = (1, 101) 25.38, *p* > 0.001) was unaffected by the context in which they were presented (F ≤ 0.54, *p* ≥ 0.655 for main effect of Context and interaction).

### 3.4. Changes in Rated Appetite 

**Figure 3:**
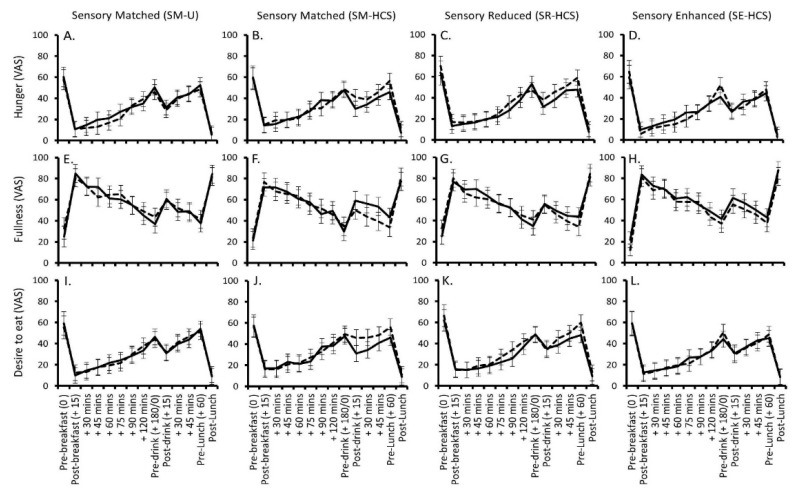
Mean (±95% CI) changes in hunger (**A**–**D**) fullness (**E**–**H**) and desire to eat (**I**–**L**) from pre-breakfast to post-lunch on the days the lower energy (dashed line) and higher energy (black line) soymilks were consumed across the four different contexts.

Changes in rated hunger, fullness and desire to eat are shown in Figure 3A–L. Data for prospective consumption and thirst followed the same pattern as hunger and desire to eat, and are not shown. There was a main effect of ‘Time’ across all of the appetite ratings (F ≥ 47.11, *p* < 0.001 for all main effects): hunger, desire to eat, prospective consumption and thirst all decreased immediately after breakfast; increased in the time between breakfast and the mid-morning drink; decreased again post-drink and then peaked again before lunch. The opposite pattern was seen in ratings of fullness.

Changes in rated hunger depended on the energy content of the beverage and the context in which it was consumed (Time × Energy Content × Context interaction: F (36, 1852 = 1.51, *p* = 0.027). Figure 3A–D suggest that hunger was higher after consuming the sensory-matched and sensory-reduced lower-calorie soymilk labelled with the HCS, specifically when they were consumed mid-morning. Moreover, hunger and desire to eat appeared to spike immediately before lunch on the day the sensory-reduced beverage was consumed. However, none of these specific post-hoc comparisons reached significance after Bonferroni corrections were applied (*p* ≥ 0.100). Similar trends were also observed in rated fullness (Figure 3E–H), desire to eat (Figure 3I–L) and prospective consumption (not shown), whereby participants tended to report feeling less full, a stronger desire to eat and greater prospective consumption after consuming the sensory-matched labelled beverage in the time after the mid-morning exposure. However, the Time × Energy Content × Context interactions were non-significant for these ratings (F ≤ 1.02, *p* ≥ 0.434). There was a significant main effect of beverage context on prospective consumption (F (3, 319) = 2.95, *p* = 0.033), which indicated that participants consuming the reduced-calorie beverage in the sensory-matched labelled condition (M = 36.5 ± 3.0) tended to have a larger prospective consumption overall, and specifically compared to participants consuming the reduced-calorie labelled beverage in the sensory-enhanced condition (M = 30.3 ± 2.9; *p* = 0.022). There were no other significant main effects or interactions between any of the study variables and the appetite ratings (F ≤ 2.22, *p* ≥ 0.086).

## 4. Discussion

In the current study, total energy intake across the day was consistently lower after consuming lower calorie beverages, regardless of how they were presented. Labelling a reduced calorie beverage as a healthier choice, in combination with strategies to match, reduce or enhance its sensory characteristics relative to an ‘original’ version had small effects on sensory and hedonic ratings and the experience of appetite. However, eating behaviours recorded over the course of the test day were unaffected. Consuming reduced-calorie products in place of original versions can generate small reductions to daily energy intake that support long-term reductions in energy intake and weight management [11,33]. If lower energy intake from reduced calorie products can be sustained over days, weeks and months, then the current data on acute energy compensation suggest that orienting a consumer to the caloric value of the product may not inhibit these effects.

Contrary to concerns about the licencing effect of consuming foods perceived to be healthier [34], awareness of consuming a lower calorie ‘healthier choice’ product did not promote increases in energy intake or greater compensatory eating at a later meal. Possible explanations include the use of non-dieting participants and a moderately healthy and familiar test product. Although participants were somewhat restrained and familiar with the Healthier Choice Symbol, they were not actively dieting, which can be an important driver of food decision making and eating behaviour [35] and potentially increase sensitivity to labelled health information. Additionally, compensatory eating might be more prominent when health labels are applied to a product initially perceived to be less (rather than more) healthy [36], when the implied benefits (e.g., “this is better for me and lower calorie”) have more scope to improve on less healthy baseline perceptions [17]. Finally, expectations about how satiating a beverage will be can impact satiety and later food intake [30], and although the HCS has been shown to increased perceived healthiness and decreased calorie estimations [19] it failed to change expectations about filling the lower calorie soymilks would be, which is likely to be another contributing factor to the lack of label effects on later food intake. It is important to acknowledge that the impact of health and nutrient-related labelling on eating behaviours will be dependent, in part, on the type of food they are applied to (e.g., healthy or indulgent), characteristics of the consumer groups they are designed for (e.g., dieting status), and their potential to shift beliefs about how satiating the product will be.

It is also possible that licencing effects do not easily extend to the aspects of eating behaviour captured in this study. Research to date has tended to focus on how health and nutrient labels can have an immediate impact on product evaluations, increased portion selections and intake of the labelled products (reviewed in [22,37]). Fewer studies have investigated how labels applied to a fixed portion of a food or beverage can impact satiety responses over time and energy intake from different foods at later meals. Of these, some have shown that labels like ‘lighter’, ‘low fat’ and ‘healthy’ can modify the experience of satiety and energy intake within a day [23,24,26] while others have not [27,38]. Beliefs generated from FOP labels may have a weaker impact on the experience of bottom-up nutrient-derived satiety and resulting eating behaviours, compared to product evaluations and portion choices that may be more reliant on top-down cognitive cues.

In support of the top-down influence of label-generated beliefs, the Healthier Choice Symbol did elicit some interesting shifts in the sensory and hedonic ratings of the products. The sensory-matched higher- and lower-calorie soymilks were equally pleasant, sweet, and creamy when the calorie difference went unlabeled. However, the same lower-calorie soymilk was perceived to be less sweet and thick when participants were informed the calorie reduction through the Healthier Choice Symbol. This is an example of assimilation [17], where the perceived sensory experience is shifted to be more in-line with the initial expectation that a healthier reduced calorie soymilk would be less sweet and thick [19], and is a potential risk factor for reduced product acceptance. Assimilation effects may also explain why the thicker and creamier characteristics of the sensory-enhanced soymilk confirmed during pilot testing were less evident in the main study when the products were evaluated in the context of the Healthier Choice Symbol. Despite these differences in sensory characteristics, overall pleasantness of the sensory-matched lower-calorie soymilks was unaffected by the health label and depended on the sensory modifications instead; pleasantness was maintained for the sensory-enhanced version that was less familiar but designed to be sweeter and creamier. By contrast, pleasantness was significantly lower for the sensory-reduced version that was designed to taste less thick, creamy and sweet. This indicates that sensory reduction was the only strategy to negatively affect the product’s hedonic value in the short term.

A final consideration is the potential for the healthier choice label to impact the experience of appetite. Participants rated feeling hungrier and less full after consuming a lower (relative to higher) calorie soymilk when the calorie reduction was made explicit with a combination of the Healthier Choice Symbol and reduced sensory characteristics. By contrast, rated appetite appeared to be unaffected when the calorie reduction was covert or consumed with thicker and creamier sensory cues. This supports the use of ‘silent’ reformulation strategies for maintaining the satiating power of reduced calorie products, but also suggests that sensory enhancements could be used to offset a possible increase in appetite when a product is explicitly presented as healthier. It is important to note, however, that differences in appetite were small and occurred only after the soymilk was consumed alone and not as part of breakfast. It is arguably harder to differentiate the specific satiating effects of individual foods or beverages when they are consumed together as part of a larger meal; therefore, participants may have had more opportunity to interpret changes in appetite in the context of the labelled calorie reduction outside of a meal setting. It is possible that lower satiating power of reduced calorie products could be attenuated by consuming these alongside other caloric items, although this idea remains to be formally tested and will require a larger sample size to achieve the power to detect consistent shifts in reported appetite.

## 5. Conclusions

Reformulation strategies to reduce the energy density of commonly consumed food and beverage products are necessary, but a better understanding of how they should be presented to consumers is required. Consistent with existing evidence, consuming a reduced calorie product in place of an ‘original’ version lowered daily energy intake as participants made little short-term adjustments to intake of other foods consumed later in the day. Awareness of consuming a lower-calorie ‘healthier choice’ beverage did not increase compensatory eating behaviours, but did impact the perceived sensory quality of the beverage and changes in appetite. If food companies or governments opt to apply labels such as the Healthier Choice Symbol to food and beverage products, then combining these with appropriate taste and texture enhancements is one strategy to maintain acceptance of reformulated ‘healthier’ products and avoid negative effects on appetite. Research considering different strategies for the presentation of reduced calorie products is necessary to understand the efficacy of reformulating foods as a population-based strategy for weight management, but should consider the impact on consumer’s appetite and eating behaviour over longer time frames, across different food contexts and different consumer groups.

## Figures and Tables

**Table 1:** Mean (±SD) participant characteristics within each of the four beverage context groups.

	SM-U (*n* = 28)	SM-HCS (*n* = 27)	SR-HCS (*n* = 28)	SE-HCS (*n* = 28)	*p*-Value ^a^
Age (years)	22.9 ± 1.9	23.2 ± 2.4	22.8 ± 1.8	22.9 ± 1.2	0.861
BMI (kg/m^2^)	22.7 ± 2.6	22.4 ± 2.3	22.1 ± 2.4	22.3 ± 2.7	0.787
Body Fat (%)	24.2 ± 8.7	23.9 ± 5.5	23.2 ± 6.1	23.5 ± 8.0	0.961
Dietary Restraint (0–21)	8.5 ± 5.3	10.7 ± 4.8	8.1 ± 5.1	8.0 ± 5.1	0.178
Consumption Frequency:					
Soymilk (per week)	1.0 ± 1.0	1.0 ± 1.3	1.7 ± 1.9	0.9 ± 0.8	0.129
Fried rice (per week)	0.9 ± 0.7	0.6 ± 0.5	0.9 ± 0.8	0.7 ± 0.5	0.284
Reported using HCS (%)	64	74	57	57	

^a^*p*-values represent the main effect of the beverage Context on each of the participant characteristics, where F (3, 107) ≤ 1.94 for all analyses.

**Table 2:** Sensory evaluations (Mean ± 95%CI) of the higher (Original) and lower (Reduced) calorie soymilks.

	SM-U (*n* = 28)		SM-HCS (*n* = 27)		SR-HCS (*n* = 28)		SE-HCS (*n* = 28)		
	Original	Reduced	Original	Reduced	Original	Reduced	Original	Reduced	Energy × Context ^a^
Soymilk									
Pleasant	65.8 ± 6.0	68.7 ± 7.1 ^ns^	76.8 ± 6.2	72.2 ± 7.3 ^ns^	66.4 ± 6.0	49.4 ± 7.1 *	73.1 ± 6.0	66.4 ± 7.1 ^ns^	0.003
Sweet	56.0 ± 6.6	54.4 ± 7.6 ^ns^	65.8 ± 6.8	53.2 ± 7.9 *	66.6 ± 6.6	24.3 ± 7.6 *	66.6 ± 6.6	63.5 ± 7.6 ^ns^	<0.001
Thick	63.6 ± 7.3	57.8 ± 7.6 ^ns^	67.4 ± 7.5	57.2 ± 7.9 *	57.7 ± 7.3	39.6 ± 7.6 *	63.0 ± 7.3	75.0 ± 7.6 ^ns^	<0.001
Creamy	63.3 ± 7.0	61.0 ± 7.6 ^ns^	65.1 ± 7.3	58.2 ± 7.9 ^ns^	59.0 ± 7.0	35.8 ± 7.6 *	65.9 ± 7.0	74.2 ± 7.6 *	<0.001
Familiar	53.5 ± 8.9	61.6 ± 9.5 ^ns^	65.6 ± 9.2	66.3 ± 9.8 ^ns^	60.6 ± 8.9	52.9 ± 9.5 ^ns^	68.3 ± 8.9	41.6 ± 9.5 *	<0.001
Filling	64.2 ± 8.1	64.2 ± 7.6	62.9 ± 8.4	59.5 ± 7.9	53.0 ± 8.1	50.4 ± 7.6	66.1 ± 8.1	71.6 ± 7.6	0.335
Lunch									
Pleasant	56.8 ± 8.0	61.0 ± 7.9	65.3 ± 8.1	70.3 ± 8.0	55.9 ± 8.0	58.6 ± 7.9	61.1 ± 8.0	72.5 ± 7.9	0.427
Familiar	50.7 ± 10.3	52.1 ± 10.0	56.0 ± 10.5	61.6 ± 10.2	56.9 ± 10.3	56.8 ± 10.0	60.7 ± 10.3	62.8 ± 10.0	0.917

^a^*p*-values represent the Sensory Context × Energy Density interaction on each of the sensory characteristics. F (3, 105) ≥ 4.80 for all significant interactions and F (3, 105) ≤ 2.52 for the two non-significant interactions. * Indicates a significant within-group Bonferroni corrected comparison (at *p* ≤ 0.06) between the lower and higher energy soymilks in each of the Sensory Context groups. ^ns^ Indicates no significant difference between the lower and higher energy soymilks in each of the groups.

## Appendix A

**Table A1:** Soymilk Recipes.

	Higher Energy ^a^	Lower Energy	Lower Energy	Lower Energy
Ingredients		Sensory Matched ^b^	Sensory Reduced ^c^	Sensory Enhanced ^d^
Soymilk (g) ^1^	250	250	250	250
Maltodextrin (g) ^2^	30	0	0	0
Sucralose (g) ^3^	0.0250	0.0350	0.0100	0.0450
Guar gum (g) ^4^	0.20	0.45	0.20	1.00
Vanilla extract (g) ^5^	0	0	0	2
Cream cheese flavour (g) ^6^	0	0	0	1
Water (g)	51	70	70	70
Total energy content (kcal)	211.1	97.5	97.1	98.4
Total Volume (mL)	315	315	315	315

^a^ Designed to mimic an original soymilk in taste, texture and appearance; ^b^ Sensory characteristics were designed to match the higher energy version; ^c^ Sensory characteristics were designed to be less sweet, thick and creamy than the higher energy version; ^d^ Sensory characteristics were designed to be thicker, sweeter and creamier than the higher energy version. ^1^ Nutrisoy No Added Sugar soymilk (F&N, Singapore); ^2^ MALTRIN^®^ M100, Singapore: DE 10-12; ^3^ Tate& Lyle, US; ^4^ Myprotein^®^, UK; ^5^ Nielsen-Massey Vanillas Inc., US; ^6^ LorAnn Oils, US.

- There were four different soymilks produced in total (see Appendix A Table A1).
- Participants in the SM-U and SM-HCS groups consumed the same lower energy sensory-matched soymilks, the only difference was whether this was presented with or without the label.
- The final recipes and sensory modifications were derived from two rounds of pilot testing with non-trained panel of participants who did not take part in the main study (*n* = 17; data not reported).
- The soymilks were refrigerated and served chilled and sealed in plastic cups with the HCS label placed directly on the plastic seal of the relevant beverage.
- The drinks were volume matched to 315 mL portions by the addition of water.

**Table A2:** Mean (±95% CI) Energy Intake (Kcal) across the Test Day Broken Down for the Fixed Breakfast, Fixed Test Drinks, Ad Libitum Lunch Intake and Food Diary Records for the Rest of the Day.

		Breakfast (kcal)	Test Beverages (kcal)	Ad Libitum Lunch Intake (Kcal + 95%CI)	Self-Reported Intake (Kcal + 95%CI)
SM-U	Original	299	422	368 ± 56	883 ± 168
	Reduced	299	196	405 ± 58	869 ± 168
SM-HCS	Original	299	422	446 ± 57	1031 ± 173
	Reduced	299	196	395 ± 56	876 ± 170
SR-HCS	Original	299	422	407 ± 56	978 ± 173
	Reduced	299	196	445 ± 58	862 ± 173
SE-HCS	Original	299	422	467 ± 57	915 ± 174
	Reduced	299	196	423 ± 56	917 ± 169
